# Effects of abiotic stresses on the expression of chitinase-like genes in *Acyrthosiphon pisum*

**DOI:** 10.3389/fphys.2022.1024136

**Published:** 2022-11-23

**Authors:** Chunchun Li, Inzamam Ul Haq, Aroosa Khurshid, Yan Tao, Peter Quandahor, Jing-Jiang Zhou, Chang-Zhong Liu

**Affiliations:** ^1^ Biocontrol Engineering Laboratory of Crop Diseases and Pests of Gansu Province, College of Plant Protection, Gansu Agricultural University, Lanzhou, China; ^2^ CSIR-Savanna Agricultural Research Institute, Tamale, Ghana; ^3^ State Key Laboratory of Green Pesticide and Agricultural Bioengineering, Guizhou University, Guiyang, China

**Keywords:** *Acyrthosiphon pisum*, chitinase, expression profiles, phylogenetic analysis, 20hydroxyecdysone

## Abstract

Insect chitinases play a crucial part to digest chitin in the exoskeleton during the molting process. However, research on insect chitinase related to the environmental stress response is very limited. This study was the first conducted to expression analysis of chitinase- related genes in *A. pisum* under abiotic stresses. Here, we identified five chitinase-like proteins (ApIDGF, ApCht3, ApCht7, ApCht10 and ApENGase), and clustered them into five groups (group II, III, V, Ⅹ, and ENGase). Developmental expression analysis revealed that the five *A. pisum* chitinase-related genes were expressed at whole developmental stages with different relative expression patterns. When aphids were exposed to various abiotic stresses including temperature, insecticide and the stress 20-hydroxyecdysone (20E), all five chitinase genes were differentially expressed in *A. pisum*. The results showed that insecticide such as imidacloprid down-regulated the expression of these five Cht-related genes. Analysis of temperature stress of *A. pisum* chitinase suggested that *ApCht7* expression was high at 10°C, which demonstrates its important role in pea aphids under low temperature. Conversely, *ApCht10* was more active under high temperature stress, as it was significantly up-regulated at 30°C. Besides, 20E enhanced *ApCht3* and *ApCht10* expression in *A. pisum*, but reduced *ApCht7* expression. These findings provide basic information and insights for the study of the role of these genes under abiotic stress, which advances our knowledge in the management of pea aphids under multiple stresses.

## 1 Introduction

Chitin is a polysaccharide of *N*-acetylglucosamine (GlcNAc) residues joined by -1, 4-glycosidic links (Merzendorfer, 2006). It is widely distributed in fungus, arthropod, nematodes, and marine organism (Rudall and Kenchington, 1973; Tharanathan and Kittur, 2003). Insect chitin is the major components of the exoskeletons. It also forms the trachea lining and the peritrophic matrix (PM), which protect insects from physical abrasion, chemical erosion, and pathogenic invasion (Merzendorfer and Zimoch, 2003). The cuticular chitin is periodically degraded by the efficient chitin degradation system secreted by epithelial cells, due to the rigid cuticle structure that restricts the growth and development of the insect (Arakane and Muthukrishnan, 2010; Moussian, 2010; Arakane et al., 2012).

Chitinase is the main enzyme involved in the degradation of chitin. Insects chitinases are present mainly in the moulting fluid and midgut to enable periodic shedding of old exoskeleton and turnover of the midgut lining, which belongs to the 18 glycosyl hydrolases family and has the potential to degrade chitin and also controls the degradation of chitin into low-molecular-weight chitooligosaccharides (Kramer and Muthukrishnan, 1997; Chen et al., 2018). Commonly, according to sequence homology and phylogenetic analysis, insects chitinases have been classified into 11 groups with different relative expression levels (Arakane and Muthukrishnan, 2010). *Nilaparvata lugens* expressed *NlCht2*, *NlIDGF*, and *NlENGase* at all stages with slight periodical changes, especially in the adult female reproductive organs, whereas *NlCht4* was highly expressed only at the adult stage in the male reproductive organs (Xi et al., 2014). In *Bactrocera dorsalis*, expressions of *BdChts*, *BdIDGF4*, and *BdIDGF6* were up-regulated from eggs to adults, *BdCht5*, *BdCht8*, and *BdCht10* were also up-regulated at larvae-pupae metamorphosis stages, whereas *BdCht11* was low regulated at all developmental stages (Liu et al., 2018).

Insect chitinase genes play a major role in the physiological process of insect growth and development, as well as their adaptation to environmental stress. *B. dorsalis* showed high heat tolerance by up-regulating *IDGF* gene (Gu et al., 2019). At 45°C for 1 h, the expression of *BdIDGF4* in *B. dorsalis* was 3.15-fold higher, compared with the control, whereas the expressions of *BdIDGF1* and *BdIDGF2* were significantly up-regulated at 40°C (Gu et al., 2019). Insect steroid hormone and 20-hydroxyecdysone (20E) influence insect development and chitinase. In *Bombyx mori*, 20E induced the up-regulation of *BmCht5* at the larval–larval and larval-pupa stage (Zhang and Zheng, 2016). The expression of chitinase genes by 20E has been reported, especially in *Manduca sexta* (Kramer et al., 1993), *Tenebrio molitor* (Royer et al., 2002), *Choristoneura fumiferana* (Zheng et al., 2003), *Locusta migratoria manilensisand* (Li et al., 2015). Collectively, these effects change the expression of the chitinase gene, thus disrupting normal chitin metabolism, which may play crucial roles in insect defense against abiotic stresses.

The pea aphid, *Acyrthosiphon pisum* Harris, is one of the major agricultural pests, which inhibits crop production not just by directly feeding on plant phloem sap but also acts as a vector to various viral diseases (Ryalls et al., 2013). Globally, pea aphid management has become a major challenge, due to its small size, high fecundity, and plasticity. Consequently, wide range of synthetic insecticides is continuously used to control of pea aphids. This type of strategy is seriously increasing environmental contamination, insecticide resistance, and endangering the health of farm operators, animals, and food consumers. Therefore, there is an urgent need to find other effective and friendly methods to control *A. pisum*. Fortunately, the completion of *A. pisum* whole genome sequence has provided an opportunity to develop new strategies with molecular tools for aphid control (International Aphid Genomics Consortium, 2010). *In silico* screening of the entire genome of *A. pisum*, detected nine genes encoding putative chitinase-like proteins, including six enzymatically active chitinases, one imaginal disc growth factor, and one endo-beta-N-acetylglucosaminidase in 2010 (Nakabachi et al., 2010). Still, only a few were revised as Cht genes after the Gene Bank database update in 2017. Moreover, Nakabachi et al. (2010) demonstrated that the expression of four and two distinct chitinase-like genes of *A. pisum* to be highly up-regulated in the embryo and the midgut, respectively, suggesting specific roles in these pea aphid tissues. However, whether there are differences in chitinase gene expression in different developmental stages of *A. pisum* has not been reported. Based on previous research, we further accurately studied on the identification of chitinase- related genes in *A. pisum* in this study. We also described gene expression patterns of the chitinase- related genes in different stages. Besides, the expression pattern with qRT-PCR of the chitinase gene on environmental stress in the development stage was investigated for the first time in *A. pisum*, and understanding the chitin degradation of the pea aphid under stress conditions can help in developing a safer molecular strategy for their control.

## 2 Materials and methods

### 2.1 Insect culture

The green pea aphid *Acyrthosiphon pisum* was cultured from a single parthenogenetic female collected from an alfalfa field in Gansu Province, China. They were maintained in the greenhouse at 20 ± 1°C, 60 ± 10% relative humidity, and a photoperiod of 16 h:8 h (L:D).

### 2.2 Gene identification and phylogenetic analysis

The chitinase genes (*Chts*) were identified by searching against the genome of *A. pisum* (Ap) (https://bipaa.genouest.org/sp/acyrthosiphon_pisum/). To compare the chitinase sequences of *A. pisum* with those of other different species and explore the evolutionary relationship of chitinase identified in *A. pisum*, the protein sequences of *Phenacoccus solenopsis* (Ps), *Anopheles gambiae* (Ag), *Bombyx mori* (Bm), *Drosophila melanogaster* (Dm), *Tribolium castaneum* (Tca), *Aphis gossypii* (Ago), *Apis mellifera* (Am), *Bactrocera dorsalis* (Bd), *Nilaparvata lugens* (Nl) were downloaded from the NCBI database and listed in Supplementary Table S1. The phylogenetic tree was generated using the Maximum Likelihood method with 1,000 bootstrap tests using the MEGA 6.0 software.

### 2.3 Domain analysis

The online ExPASy Proteomics website (https://web.expasy.org/protparam/) was used to predict the theoretical parameters of the proteins. The domain architecture and signal peptide were identified from the protein sequences using the Pfam (http://pfam.wustl.edu/) and SMART search. The exon-intron organization of chitinase-like genes were predicted by sequence comparison between genomic sequences and putative cDNA sequences.

### 2.4 Sample collection at different developmental stages

To clarify the development time required for different instar stages of *A. pisum*, we observed the entire developmental stages of the pea aphid under our laboratory condition in pre-experiments. Based on the pre-experiment information, ten wingless adult aphids from the same cultural batch were inoculated on the broad bean leaves in a 9 cm diameter Petri dish and then removed after 6 h of reproduction. These primiparous aphids were allowed to grow on the broad bean leaves, and then the 1^st^-, 2^nd^-, 3^rd^-, 4^th^-instar nymphs and adults were sampled. The broad bean leaves used for the experiments were replaced after every 3 days. The sample collection process was independently repeated three times, and all samples were placed in liquid nitrogen immediately after collection and then stored at −80°C.

### 2.5 Stress treatments

#### 2.5.1 Temperature treatment

The primiparous aphids were inoculated on the leaves until they reached 2^nd^ instars. Four hundred fifty 2^nd^ instar nymphs were randomly selected and transferred to new Petri dishes. These selected nymphs were divided into 3 groups (150 per group) and reared at different temperatures in three incubators as “low temperature group (10°C)”, “normal temperature group (20°C)” and “high temperature group (30°C)” (Du et al., 2015) at 60 ± 10% relative humidity and a photoperiod of 16L: 8D. Ten aphids were randomly collected at 12 h, 24 h, 36 h, and 48 h from each temperature group as experimental samples and placed in a 1.5 ml sterile centrifuge tube. These samples were immediately frozen in liquid nitrogen and stored at −80°C. This collection procedure was repeated 3 times, so there were 360 2^nd^ instar nymphs were used in the temperature treatment experiment.

#### 2.5.2 Insecticide treatment

The leaf dipping method was used in this experiment (Ministry of Agriculture of the PRC, 2008). Newly emerged and healthy wingless adult aphids from the same batch were used in the insecticide treatments. Based on the LC_50_ results determined in the previous study (Wang et al., 2014), imidacloprid (Shanghai Yuelian Chemical Co., Ltd. China) was dissolved in double distilled H_2_O at 5.34 mg/L as the working solution. The broad bean leaves with 60 wingless adult aphids were immersed in the working solution for 10 s and placed on filter paper in a 9 cm petri dish for natural air drying, and then kept for 24 h and 48 h in the condition of 20 ± 1°C, a light cycle of L:D = 16 h:8 h, and relative humidity of RH 60 ± 10%. Adult aphids treated with clean water were used as the control group. After that, five live adults were randomly selected from each treated group (24 h and 48 h) and the control group and placed separately in three 1.5 ml sterile centrifuge tubes. The samples were immediately frozen in liquid nitrogen and stored in a refrigerator at −80°C for later use. Three biological replicates were set for each treatment group and the control group.

#### 2.5.3 20E treatment

The 20E (20-hydroxyecdysone) (Sigma Co., St Louis, MO, United States) was dissolved in 95% ethanol as the stock solution. It was diluted to 12 mg/mL with double distilled H_2_O and used as the working solution (Ma et al., 2021). Newly emerged (<6 h) nymphs were placed into 9 cm Petri dishes and fed on the leaves with their back facing up and moist absorbent cotton around the petioles until they reached the 2^nd^ instar stage. Fifty 2^nd^ instar nymphs were collected and soaked in the 20E working solution for 10 min. Similarly, fresh broad bean leaves were also treated with the 20E working solution. Then the nymphs and the treated leaves were placed on sterile filter paper to dry. The nymphs were inoculated on the treated leaves (moist absorbent cotton around the petioles) in Petri dishes at 20 ± 1°C, a light cycle of L:D = 16 h: 8 h, and the relative humidity of 60 ± 10%. The nymphs treated with 5% alcohol were used as the control group. Ten aphids were randomly sampled at 24 h, 48 h, and 72 h after the treatments from each treatment group and the control group, and placed separately in a 1.5 ml sterile centrifuge tube. The samples were immediately frozen in liquid nitrogen and stored in a refrigerator at -80°C for later use. Three independent repeats were set in the treatment group and the control group.

### 2.6 Total RNA extraction and cDNA synthesis

The total RNA of the *A. pisum* samples from different developmental stages and the 2^nd^ instar nymphs under each emergency stress (temperatures, insecticide and 20E) were isolated using TRIzol reagent (Invitrogen, Carlsbad, CA, United States) as recommended by the manufacturer. The RNA purity was determined by measuring the absorbance ratio 260/280. The gDNA was removed with the RQ1 RNase-Free DNase kit (Promega, United States), and the first-strand complementary DNA (cDNA) was synthesized using the PrimerScript RT Reagent Kit (TAKARA, Dalian, China) with 1 μg of total RNA template in a 20 μL reaction following the manufacturer’s protocol. The synthesized cDNA was stored at −20°C.

### 2.7 Real-time quantitative reverse transcription-PCR

Primer 3.0 (http://bioinfo.cu.ee/primer3-0.4.0) was used to design gene specific primers (Supplementary Table S2) and synthesized by Qinke Biotech (Beijing, China). All RT-qPCR was carried out in a 10 μL reaction mixture consisting of 5 μL of 2×SuperReal PreMix Plus (Tiangen, Beijing, China), 0.5 μL of each primer (0.2 mM), 0.5 μL of cDNA template, 0.2 μL of 50 × ROX Reference DyeΔ, and 3.3 μL of nuclease-free water. The real-time qPCR program consisted of an initial denaturation step at 95°C for 15 s followed by 40 cycles at 95°C for 10 s and 60°C for 30 s. Two reference genes, the *Elongation factor 1 alpha* (*EF1α*) and *Ribosomal protein S20* (*RPS20*) (Supplementary Table S2), were used to normalize the expression levels of targeted genes through qBase+ (Hellemans et al., 2007) based on the 2^−ΔΔCT^ method (Pfaffl, 2001). The relative expression level of Cht-related genes was presented as mean ± SE of three biological replicates.

### 2.8 Statistical analyses

Parameters measured in different stages and under abiotic stresses (temperature, Insecticide and 20E) were analysed using one-way analysis of variance (one-way ANOVA), followed by Tukey’s HSD tests to detect statistically significant differences (*p* < 0.05) between these parameters using SPSS 22.0 software.

## 3 Results

### 3.1 Identification and characterization of chitinase genes in *A. pisum*

Five chitinase genes were identified from the *A. pisum* genome, and their basic information are listed in Table 1. Among the 5 Cht-related genes, 3 genes encode for chitinases. The protein length of *ApCht3*, *ApCht7* and *ApCht10* is 473 aa, 998 aa and 2,274 aa, respectively, with the predicted MW and pI of 53.52 and 5.84, 112.75 and 6.64, 257.99 and 6.76, respectively. One *ApIDGF* (Imaginal disc growth factor) belongs to GH18 Cht-like superfamily, and encodes for a protein with 442 aa, predicted MW of 48.65 kDa and theoretical pI of 8.66. One *ApENGase* (Endobeta-N-acetylglucosaminidase) gene belongs to the glycosyl hydrolase family 85 (GH85) with deduced amino acids of 528 aa, and theoretical pI of 5.49 (Table 1).

**TABLE 1:** List of the chitinase genes characterization in the *Acyrthosiphon pisum* genome.

Gene name	Gene IDs	RefSeq mRNA	Protein length (aa)	MW (kDa)^1^	pI^1^
*ApIDGF*	LOC100160032	NM_001168671	442	48.65	8.66
*ApCht3*	LOC100169240	X0M_001952683	473	53.52	5.84
*ApCht7*	LOC100165452	XM_001950345	998	112.75	6.64
*ApCht10*	LOC100169480	XM_001943003	2,274	257.99	6.76
*ApENGase*	LOC100168559	XM_001949910	528	60.75	5.49

^1^ Mw, molecular weight; pI, isoelectric point.

**FIGURE 1:**
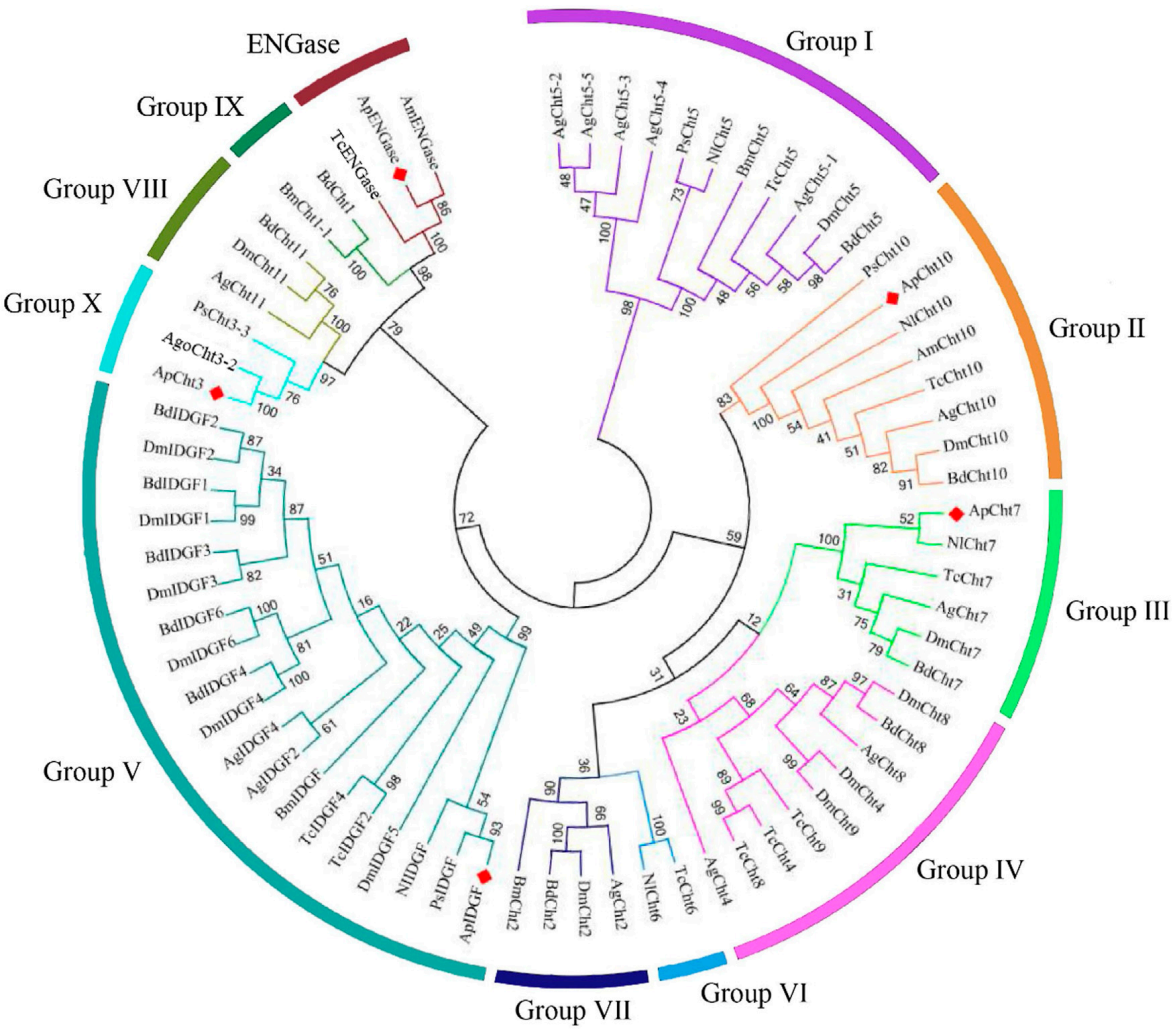
Phylogenetic analysis of chitinase genes from 10 different insects. *Acyrthosiphon pisum* (Ap), *Anopheles gambiae* (Ag), *Drosophila melanogaster* (Dm), *Tribolium castaneum* (Tc), *Bombyx mori* (Bm), *Phenacoccus solenopsis* (Ps), *Aphis gossypii* (Ago), *Apis mellifera* (Am), *Bactrocera dorsalis* (Bd), *Nilaparvata lugens* (Nl).

The chitinase from *A. pisum* and other nine insect species were used to construct a phylogenetic tree based on their protein sequence (Figure 1). The phylogenetic analysis showed ApCht10, ApCht7, ApIDGF, ApCht3*,* and ApENGase belong to groups II, III, V, Ⅹ, and ENGase, respectively. ApCht3 had a higher homology with AgoCht3-2 (100%) and PsCht3 (76%). Both ApCht10 had a higher homology with the genes of PsCht10 (83%) and NlCht10 (100%). ApIDGF also shared higher homology with PsIDGF (93%). ApCht7 shared higher homology with NlCht7 (52%). Besidess, ApENGase shared high similarities with TcENGase (100%) and AmENGase (86%).

### 3.2 Gene structure of chitinase in *A. pisum*

**FIGURE 2:**
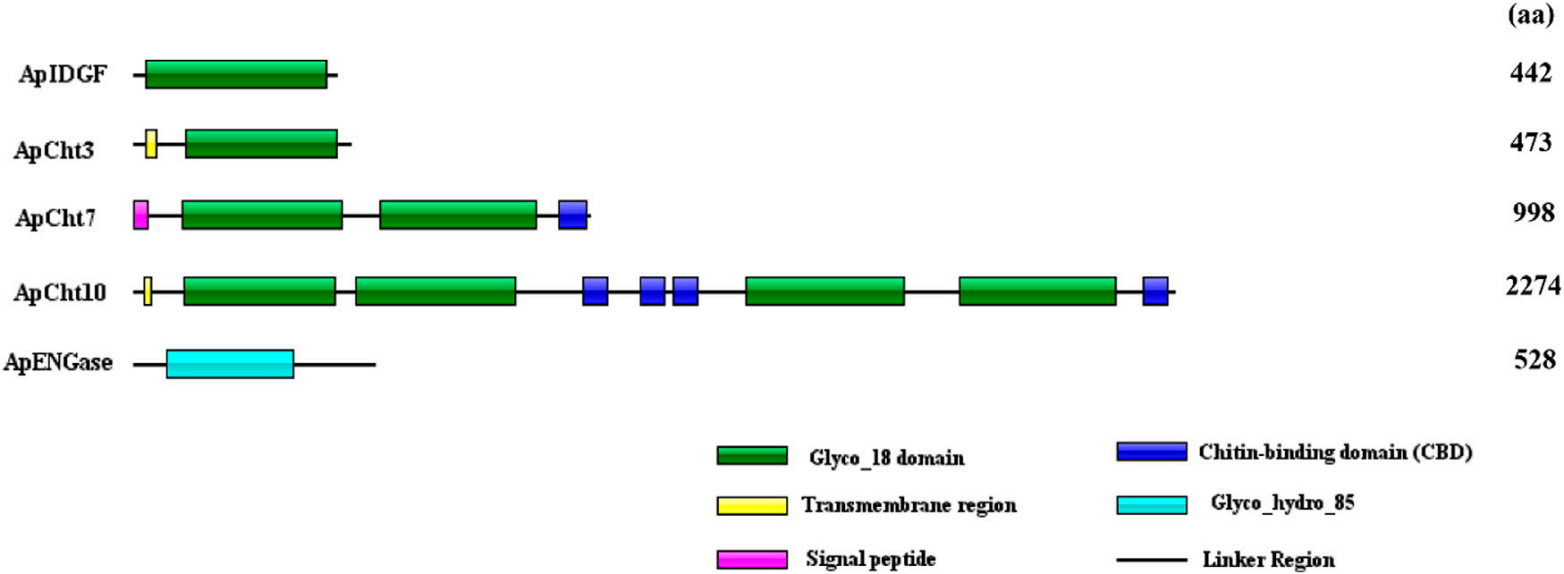
Domain analysis of 5 chitinase genes in *A. pisum*. The deduced amino acid sequences and domain architectures of 5 *A. pisum* chitinase genes were predicted by SMART software.

The domain architectures of the predicted chitinases in *A. pisum* are shown in Figure 2. All chitinases contain the catalytic domain of insect chitinases. Among them, ApIDGF and ApCht3 have one copy of the GH18 chitinase-like domain, ApCht7 and ApCht10 have two and four copies of this domain, respectively. Whereas ApENGase has a single copy of the GH85 domain (Figure 2). Multiple sequence alignments showed that each of these 8 catalytic regions had four conserved mofits with the sequences KxxxxxGGW (Mofit Ⅰ), FDGxDLDWEYP (Mofit Ⅱ), MxYDxxG (Mofit Ⅲ), and GxxxWxxDxDD (Mofit Ⅳ), respectively (Supplementary Figure S1). The conserved sequence in Mofit Ⅱ, DWEYP, is an essential characteristic of a putative chitinase. In ApIDGF and ApENGase, most of the amino acids in the four domains are different from the corresponding four conserved mofits, suggesting all four regions were poorly conserved (Supplementary Figure S1). Moreover, ApCht7 and ApCht10 have one and four chitin-binding domains (CBD), respectively, while other three genes have no CBD. Besides, one transmembrane region was detected in ApCht3 and ApCht10. One signal peptide was detected in ApCht7 (Figure 2).

**FIGURE 3:**
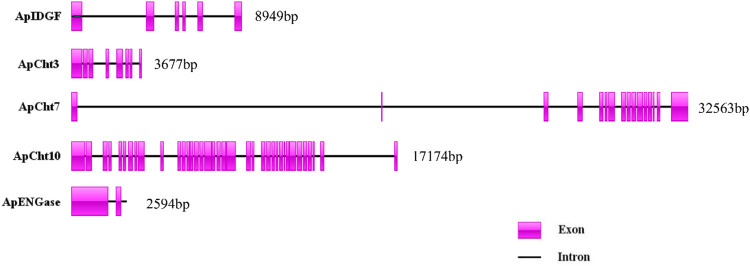
Exon-intron organization in 5 chitinase genes in *A. pisum*. The exons Exons are displayed as red box areas. The lines between the boxes represent introns.

The exon/intron organization of 5 chitinase genes within the *A. pisum* genome is shown in Figure 3. There is a clearly diverged organization among the chitinase genes in the number of exons-introns and the gene sizes. *ApIDGF*, *ApCht3,* and *ApENGase* had six, eight, and two exons, whereas *ApCht7* and *ApCht10* had 16 and 37 exons respectively. The sizes of their introns ranged from 57 bp to 3,381 bp (Supplementary Table S3).

### 3.3 Developmental expression patterns of chitinase genes

**FIGURE 4:**
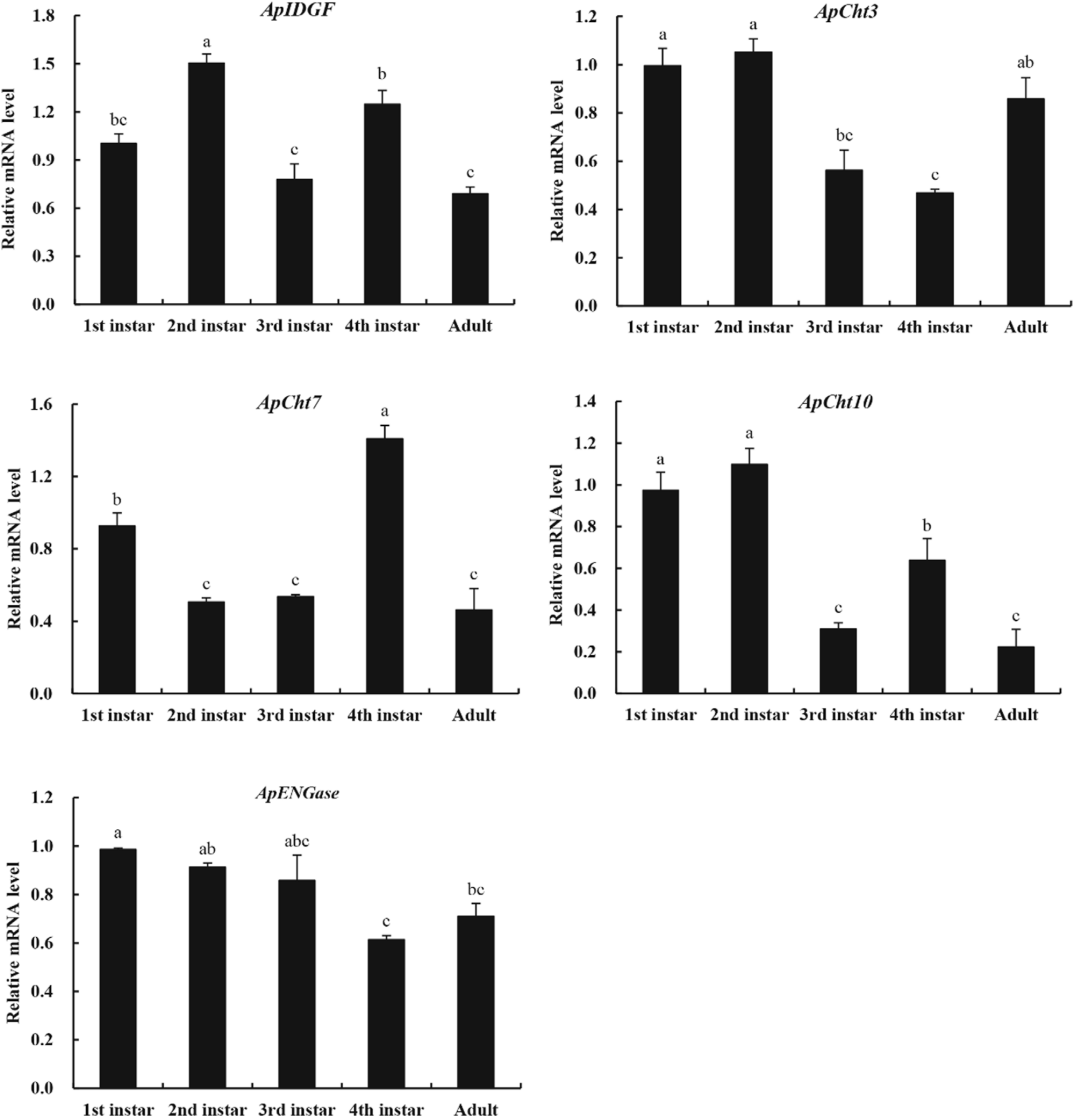
Developmental expression patterns of chitinase genes. Different letters on the top of the columns indicate significance in the difference of expression levels by Tukey’s HSD test (*p <* 0.05).

We used RT-qPCR to examine the specific expression patterns of chitinase genes at different developmental stages (1^st^-, 2^nd^-, 3^rd^-, 4^th^-nymph and adults) (Figure 4). *ApCht10* was mainly expressed in 1^st^- and 2^nd^-instar nymphs. *ApCht7* expression was abruptly increased at the 4^th^ instar stage and then decreased until adult stage. The expressions of *ApIDGF* and *ApCht10* had similar patterns, and were significantly higher at the 4^th^ instar stage than at the 3^rd^ instar and adult stages (*p* < 0.05). The expression level of *ApENGase* was stable at all stages. Moreover, *ApCht3* had a special expression pattern that was confined to the adult stage and was increased by a great margin in the adult stage relative to those of other chitinase genes (Figure 4).

### 3.4 Effect of temperature stress on the expression of the chitinase genes

**FIGURE 5:**
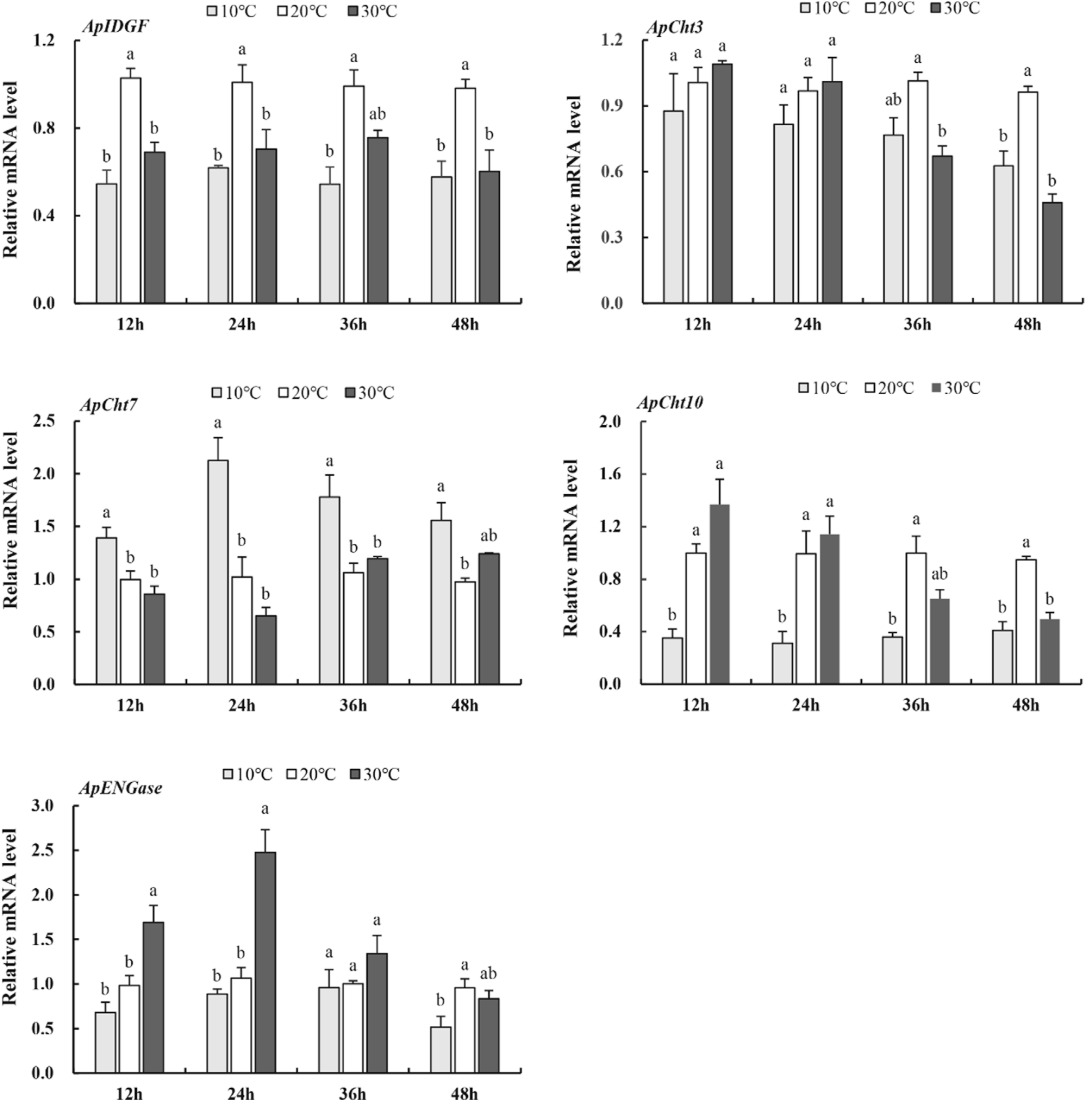
Expression of the chitinase genes in response to temperature stress. Different letters on the top of the columns indicate significance in the difference of expression levels by Tukey‘s HSD test at same treatment time (*p <* 0.05).

To examine the expression of these chitinase genes in response to temperatures, the *A. pisum* at the 2^nd^ instar nymph stage were treated at 10°C, 20°C, and 30°C for various periods. At 10°C, the expression of *ApCht3*, *ApCht10*, *ApIDGF*, and *ApENGase* was lower than those at 20°C (Figure 5). While the expression of *ApC*ht7 was higher by 0.4-, 1.1-, 0.7- and 0.6-fold at 12 h, 24 h, 36 h and 48 h at 10°C than at 20°C (*p* < 0.05). At 30°C, the expression of *ApIDGF* was significantly reduced by 0.3 to 0.6-fold relative to those at 20°C (*p* < 0.05). Similarly, the expression of *ApCht7* was reduced at 12 h and 24 h at 30°C, but it gradually increased with the treatment time and peaked at 48 h with a 1.3-fold higher at 30°C than that at 20°C (*p* < 0.05) (Figure 5). *ApCht3* and *ApCht10* had contrary expression patterns to *ApCht7*, the treatment at 30°C for 12 h and 24 h increased their expression but the treatment for 48 h decreased their expression by1.1-fold for *ApCht3* and 0.9-fold for *ApCht10* than their expression under the 20°C treatment (*p* < 0.05). The *ApENGase* expression was up-regulated by the 10°C treatment at 12 h and 24 h, but decreased with the extension of exposure time, and reached the lowest level at 48 h (Figure 5; Supplementary Figure S2).

### 3.5 Effect of insecticide stress on the expression of the cht-related genes

**FIGURE 6:**
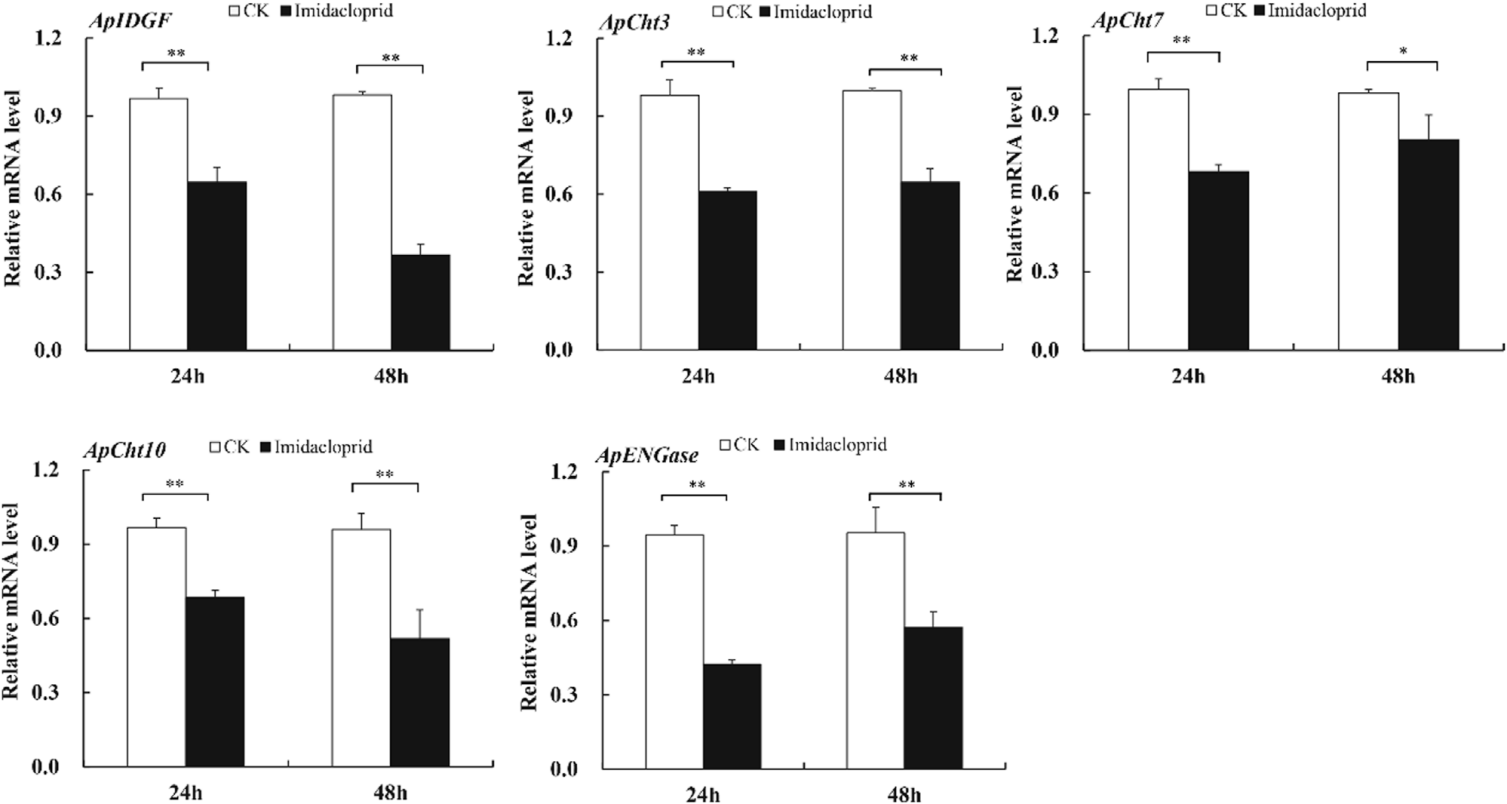
Expression of the Cht-related genes in response to insecticide stress. Different letters on the top of the columns indicate significance in the difference of expression levels by Tukey‘s HSD test at same treatment time (*p < 0.05 and **p < 0.01 compared with the control group).

The expression of chitinase-related genes was significantly reduced by the imidacloprid treatment relative to that of the control (Figure 6). Furthermore, the expression of *ApIDGF* and *ApCht10* gradually reduced by 0.8 and 0.3-fold at 48 h than that of 24 h, respectively (*p <* 0.05) (Figure 6). The imidacloprid treatment also significantly reduced the expression of *ApCht3* during 24 h and 48 h relative to the control treatment, however, stable expression levels were observed during this time under the imidacloprid treatment (Figure 6). The expression of *ApCht7* and *ApENGase* was increased by the imidacloprid treatment with prolonged time, and significantly lower than that of the control (*p* < 0.05) (Figure 6; Supplementary Figure S3).

### 3.6 Effect of 20E treatment on the expression of the cht-related genes

**FIGURE 7:**
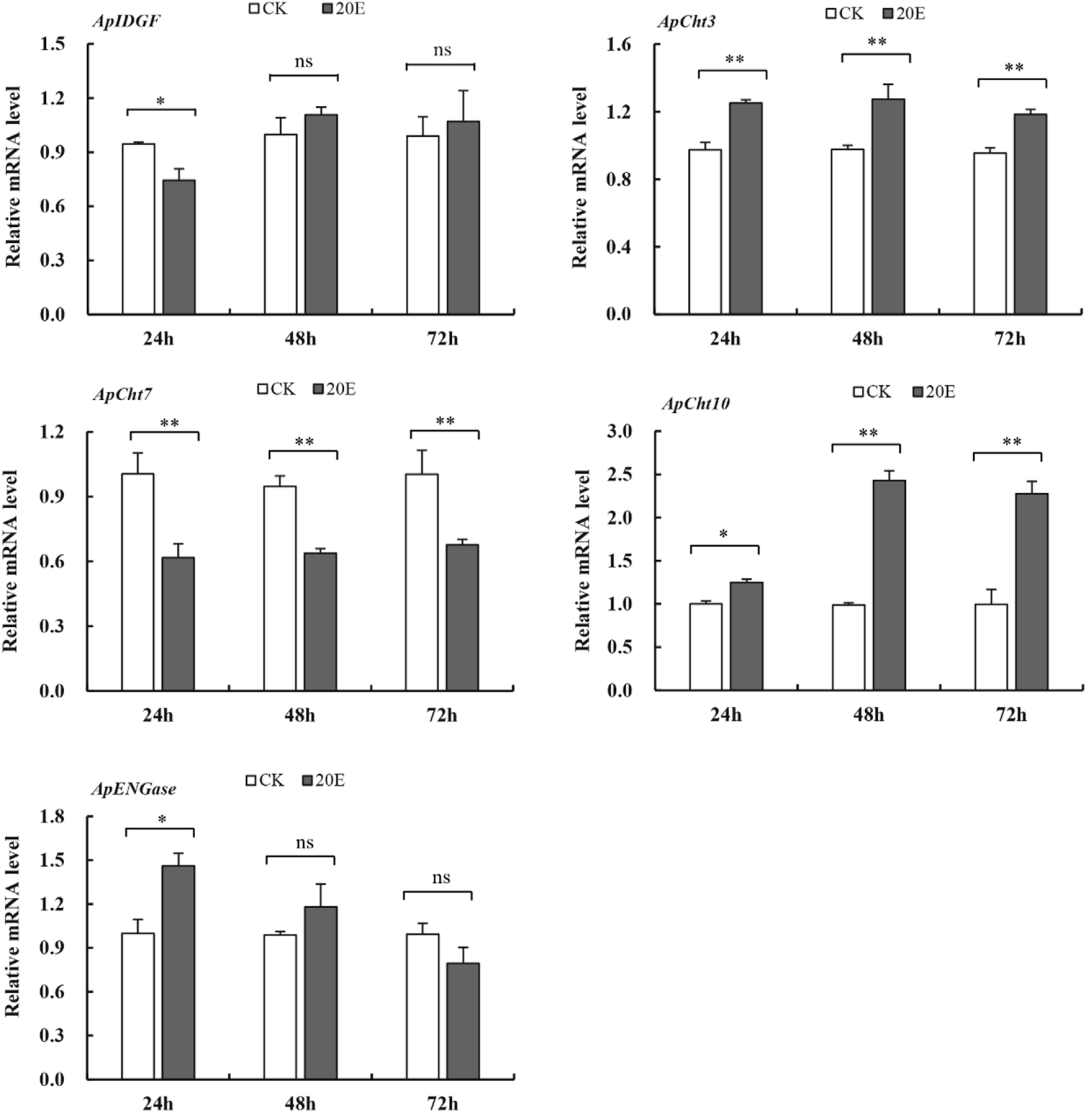
Expression of the Cht-related genes in response to 20E treatment. Different letters on the top of the columns indicate significance in the difference of expression levels by Tukey’s HSD test at same treatment time (*p < 0.05 and **p < 0.01 compared with the control group).

The 20E treatment upregulated the expression of *ApCht3* and *ApCht10* at 24 h compared with the control, and the highest expression occurred at 48 h (Figure 7). The 20E treatment significantly reduced *ApCht7* expression by between 0.63-fold and 0.48-fold compared with the control (*p* < 0.05). The 20E treatment for 24 h inhibited the expression of *ApIDGF*, but as time prolonged after 48 h the expression of *ApIDGF* was gradually increased by 0.1- and 0.8-fold than that of the control at 48 h and 72 h, respectively (*p* > 0.05). Contrary to *ApIDGF*, the expression of *ApENGase* gradually declined as the treatment time was prolonged (Supplementary Figure S4). Still, its expression level was higher than that of the control at 24 h and 48 h under the 20E treatment (Figure 7).

## 4 Discussion

Identification of Cht-related genes by bioinformatic approaches is valid and reliable due to the publications of insect whole genome sequences. This study identified five chitinases and chitinase-like proteins genes from the *A. pisum* genome. Among these, three *Chts* genes were encoded true chitinases and closely related to those chitinases with chitinolytic activities, while the remaining two genes appeared to encode an *IDGF* and an *ENGase*, respectively. The previous report also identified 9 Cht-related genes in *A. pisum* (Nakabachi et al., 2010). However, four of those Cht-related genes (IDs: LOC100160065, LOC100169337, LOC100164767, and LOC100162732) were not identified in the present study. Moreover, *A. pisum* has fewer chitinase-like genes as reported for other hemimetabolous insects such as *N. lugens* and *P. solenopsis* (Xi et al., 2014; Omar et al., 2019) than insect species especially Coleoptera and Diptera. This was speculated as the expansion of chitinase genes in dipteran and coleopteran insects (Nakabachi et al., 2010).

Based on the similarities in amino acids and phylogenetic analysis, the classification of insect Chts and Cht-like proteins has been increased from 8 groups (I-VIII) to 11 groups (I-X and H), which can provide consistent classification and nomenclature of chitinase and chitinase-like genes in different insect species (Arakane and Muthukrishnan, 2010; Tetreau et al., 2015). Compared with previous results from *A. pisum* (Nakabachi et al., 2010), we have determined that the previously assigned four genes previously named *ApCht1*, *ApCht2*, *ApCht4*, and *ApCht6*, were renamed as *ApIDGF*, *ApCht10*, *ApCht7*, and *ApCht3*, and all of the five chitinase and chitinase-like proteins were identified in group II (ApCht10), III (ApCht7), V (ApIDGF), Ⅹ (ApCht3) and ENGase (ApENGase), respectively. Among them, ApENGase is closely related to *A. mellifera* and *T. castaneum* ENGase, and the other four chitinases have very high homology with Chts of *P. solenopsis* and *N. lugens*. Previous studies have shown that there are four conserved regions in the amino acid sequence of the catalytic domain of insect chitinase GH18. The conserved sequence in Mofit II, DWEYP, is an essential characteristic of a putative chitinase, and the residue E is a putative protein donor essential for catalytic activity (Zhu et al., 2008a). While the Mofit II regions were observed to be poorly conserved in ApIDGF and ApENGase, indicating that these proteins lack chitinase activity. Although IDGF lacks chitinase activity. They may be used as a receptor binding to a cell surface (Kawamura et al., 1999; Bryant, 2001; Varela et al., 2002), and indispensable for adult eclosion (Zhu et al., 2008b). ApCht10 is the longest chitinase gene in the pea aphid and has 4 catalytic domains and 4 CBDs identical to that of *B. dorsalis* (Liu et al., 2018). ApCht7 has two catalytic domains and one CBD, and ApCht3 contains one catalytic domain. An interesting finding is that chitin-binding domains can bind chitinase to insoluble substrates with a high degradation efficiency (Arakane et al., 2003). Our data show that the domain II is well conserved in ApCht3, ApCht7, and ApCht10, suggesting they all have chitinase activity. ApCht7 and ApCht10 also have one and four chitin-binding domains respectively, which further shows that ApCht7 and ApCht10 have the degradation activity of chitinase.

The studies on the expression profiles of chitinase-related genes in the pea aphid at different developmental stages mainly focus on chitin synthesis pathway genes (Wang et al., 2021a; Wang et al.,2021b; Ye et al., 2019), but there are relatively few studies on chitin metabolism- related genes. Insect chitinases not only play a very important function in the degradation of old exoskeleton and turnover of the gut lining, but are also involve in the formation of barrier tissues, detoxification and immunity (Pesch et al., 2016; Broz et al., 2017), and these genes showed specific developmental expression pattern in insects. In *A. gambiae*, the study of stage-specific gene expression showed that most chitinase genes were expressed at the larval stages, while *AgCht8* was mainly expressed at pupal and adult stages (Zhang et al., 2011). In this study, real-time qPCR demonstrated that the five *A. pisum* chitinase-related genes were expressed at all developmental stages with different relative expression patterns. This result fully reflects the diversity and complexity of the chitinase regulation mechanism. Previous research has shown that Group I and II chitinases are involved in molting by digesting cuticular chitin, whereas Group III chitinases have a morphogenetic role in insect development (Zhu et al., 2008b). In the present study, the group II *ApCht10* showed a high expression level at both the first and second instar nymph stages in *A. pisum*, which is a similar to *P. solenopsis* (Omar et al., 2019) and *B. dorsalis* (Liu et al., 2018). This indicates that *Cht10* probably play an active role at the early developmental stages of *A. pisum*. Conversely, the group III gene *ApCht7* was highly expressed in the fourth instar nymph, suggesting that *ApCht7* may be involved in the process of insect emergence and molting. The expression of *Cht7* was upregulated during pupa-adult molts of *Drosophila meloganster* (Pesch et al., 2016) and significantly changed at the molting stage in *Sogatella furcifera* (Chen et al., 2017) and *Mythimna separata* (Yang and Fan, 2018).

The development and growth of insects are greatly affected by environmental temperatures due to their poor ability to adjust and maintain their body temperature as poikilotherms (Hu et al., 2014). Research has found that the inadequate environment of high temperature leads to an increased expression of *IDGF4* in *B. dorsalis*, and reveal its involvement in heat tolerance (Gu et al., 2019). In the desert beetle *Microdera punctipennis*, low temperature such as 4°C and −4°C both upregulate the expression of six chitinase genes, including *IDGF2* (Lu et al., 2014). We showed the different results; that both high and low temperature can down-regulate the expression of *ApIDGF*, which indicates that this gene was negatively regulated under temperature stress in *A. pisum*. Meanwhile, the expression of *ApCht3* and *ApCht10* was also significantly decreased at 36 h under the high temperature (30°C). Noticeably, the expression level of *ApCht7* was increased at 10°C than to 20°C compared to the other genes, which is similar to the study on *DcCht6* in *Diaphorina citri* (Liu, 2021). The increasing expression pattern of *ApCht7* might reveal its involvement in low temperature. In contrast, the expression of *ApCht10* was lower at 10°C than 20°C. This indicates that *ApCht7* and *ApCht10* are more sensitive to 10°C rather than 30°C. Interestingly, Liu found that CBD in chitinase gene interacts with heat shock chaperone in response to low temperature stress (Liu, 2021). *ApCht7* and *ApCht10* have one and four CBD, respectively, which may play a role in the responses to the low temperature.

Neonicotinoid imidacloprid are nicotinic acetylcholine receptor agonists which disrupt the function of insect neurons and cause paralysis and death (Bebane et al., 2019). Imidacloprid is the most effective insecticides for controlling pea aphids. The previously reported the fecundity and longevity were affected significantly when green pea aphids were exposed to sublethal doses of imidacloprid (Wang et al., 2014). Using the same insect, insecticide, and doses, here we showed that the down-regulation the expression of all the five chitinase genes when *A. pisum* were exposed to sublethal concentrations of imidacloprid. Similarly, Liu found that the expressions of *BdIDGF1*, *BdIDGF2* and *BdCht7* were significantly down-regulated compared with the control group when the adults of *B. dorsalis* were treated with the insecticide malathion (Liu et al., 2018). These results suggest that these insecticides could disturb the metabolic balance of chitin in addition to their actions on nerve systems, this may have been associated with reduced fecundity/reproduction in *A. pisum*.

Moreover, 20E is an insect hormone and has been reported to contribute to many insect physiological processes including molting (Jindra et al., 2013). Treatment with 20E enhanced the transcription level of *BdCht2* in *B. dorsalis* (Yang et al., 2013) and *MsCht5* in *M. sexta* (Kramer et al., 1993). In the present study, the 20E treatment up-regulated *ApCht3* and *ApCht10* expression over a prolonged time. Similarly, the expression of *TmCht10* gene in *Tenebrio molitor* was significantly upregulated with 20E treatment (Royer et al., 2002). Whereas 20E significantly reduced *ApCht7* expression compared with the control. This indicates that *ApCht7* is negatively regulated by 20E, which is analogous to chitinases in *M. separata*, which was reported that 20E could induce the expression of *MsCht7* of *M. separata*, and advance the molting time (Yang and Fan, 2018). Our results indicate that the expression levels of chitinase genes can be induced by 20E in pea aphids.

In conclusion, we identified five genes encoding Cht-related proteins in *A. pisu*m with different expression patterns during development and under stress. The differential expression of these chitinase genes in response to stresses confirms that each gene may function differently under stress conditions. The developmental-specific and stress-inducible expressions suggest that the insect chitinases may have diverse functions and play roles in response to environmental stress. We therefore speculate that *A. pisum* chitinases may be one of the potential signal molecules in the cross tolerance and cross-talk to a changing environment.

## Data Availability

The original contributions presented in the study are included in the article/Supplementary material, further inquiries can be directed to the corresponding author.
