# Supplementary material for: Effects of abiotic stresses on the expression of chitinase-like genes in Acyrthosiphon pisum
Source: Front Physiol. 2022 Nov 23;13:1024136. doi: 10.3389/fphys.2022.1024136 (PMC9727142; doi:10.3389/fphys.2022.1024136)

Supplementary Table 1. Information of insect chitinase proteins for phylogenetic tree construction

Species	Protein	Accession number	Phylogenetic group
Anopheles gambiae	*AgCht2*	XP_315650	Diptera
	AgCht4	XP_315351	Diptera
	AgCht5-1	HQ_456129	Diptera
	AgCht5-2	HQ_456130	Diptera
	AgCht5-3	HQ_456131	Diptera
	AgCht5-4	HQ_456132	Diptera
	AgCht5-5	HQ_456133	Diptera
	AgCht7	XP_308858	Diptera
	AgCht8	XP_316448	Diptera
	AgCht10	XP_001238192	Diptera
	AgCht11	XP_310662	Diptera
	AgIDGF2	XP_001237925	Diptera
	AgIDGF4	XP_317398	Diptera
Drosophila melanogaster	*DmCht2*	NP_477298	Diptera
	DmCht4	NP_524962	Diptera
	DmCht5	NP_650314	Diptera
	DmCht7	NP_647768	Diptera
	DmCht8	NP_611542	Diptera
	DmCht9	NP_611543	Diptera
	DmCht10	EAA46011	Diptera
	DmCht11	NP_572361	Diptera
	DmIDGF1	NP_477258	Diptera
	DmIDGF2	NP_477257	Diptera
	DmIDGF3	NP_723967	Diptera
	DmIDGF4	NP_727374	Diptera
	DmIDGF5	NP_611321	Diptera
	DmIDGF6	NP_477081	Diptera
Tribolium castaneum	*TcCht4*	NP_001073567	Coleoptera
	TcCht5	NP_001034524	Coleoptera
	TcCht6	XP_967813	Coleoptera
	TcCht7	NP_001036035	Coleoptera
	TcCht8	NP_001038094	Coleoptera

	TcCht9	NP_001038096	Coleoptera
	TcCht10	NP_001036067	Coleoptera
	TcIDGF2	NP_001038092	Coleoptera
	TcIDGF4	NP_001038091	Coleoptera
	TcENGase	XP_969648.1	Coleoptera
Bombyx mori	*BmCht1-1*	XP_004931749	Lepidoptera
	BmCht2	XP_004933352	Lepidoptera
	BmCht5	AAB47538	Lepidoptera
	BmIDGF	NP_001036847	Lepidoptera
Phenacoccus solenopsis	*PsCht3-3*	MH686272	Hemiptera
	PsCht5	MH686266	Hemiptera
	PsCht10	MH686270	Hemiptera
	PsIDGF	MH686273	Hemiptera
Aphis gossypii	*AgoCht3-2*	KAF0755855	Hemiptera
Apis mellifera	*AmCht10*	XP_026299805	Hymenoptera
	AmENGase	XP_001121069	Hymenoptera
Bactrocera dorsalis	*BdCht1*	MF926351	Diptera
	BdCht2	KF289944	Diptera
	BdCht5	KY681041	Diptera
	BdCht7	KY681042	Diptera
	BdCht8	KY426795	Diptera
	BdCht10	MK518061	Diptera
	BdCht11	KY426794	Diptera
	BdIDGF1	KY681043	Diptera
	BdIDGF2	KY681044	Diptera
	BdIDGF3	KY681045	Diptera
	BdIDGF4	KY681046	Diptera
	BdIDGF6	KY426796	Diptera
Nilaparvata lugens	*NlCht5*	KM217113	Hemiptera
	NlCht6	KM217114	Hemiptera
	NlCht7	KM217115	Hemiptera
	NlCht10	KM217118	Hemiptera
	NIIDGF	KM217119	Hemiptera
Acyrthosiphon pisum	*ApCht3*	XM_001952683	Hemiptera

	ApCht7	XM_001950345	Hemiptera
	ApCht10	XM_001943003	Hemiptera
	ApIDGF	NM_001168671	Hemiptera
	ApENGase	XM_001949910	Hemiptera

Supplementary Table 2. Primers for qPCR the genes of chitinase in *Acyrthosiphon pisum*

Genes	Primers	Nucleotide sequence (5′-3′)
ApIDGF	*ApIDGF*-F	GGGTATCTCTACGTACGGCC
	ApIDGF-R	AAGGGTGAGAGATGTGGTGG
ApCht3	*ApCht3*-F	CGGCAAGGACGGTTTTGTAA
	ApCht3-R	GAAAATACCATGGCGCCTCC
ApCht7	*ApCht7*-F	TACCTGAAGACATCGACCCG
	ApCht7-R	AACTTTTGTGTGCCGAACGA
ApCht10	*ApCht10*-F	ACTGGTCCAAGATCCACTGG
	ApCht10-R	GATACGTTTCGCAGCCACAT
ApENGase	*ApENGase*-F	ATGTTGACGGTGAAGCAGTT
	ApENGase-R	TCCCTGAATGCCAAACTCCA
EF1α	*EF1α*-F	CTGTGCTTATTGTCGCTGCT
	EF1α-R	TCGCTGTATGGTGGTTCAGT
RPS20	*RPS20*-F	AAGTGTGTGCTCCGAGATGA
	RPS20-R	CAGCAATGACACCGGGTTC

Supplementary Table 3. Lengths of each exon and intron of Chitinase gene in *Acyrthosiphon pisum*

基因<br>Gene	类别<br>Structure	从 5’到 3’长度<br>From 5’to 3’Length (5’-3’)
ApIDGF	Exon	553, 400, 171, 133, 269, 345.
	Intron	3381, 1134, 223, 644,1701.
ApCht3	Exon	548, 215, 224, 136, 271, 166, 103, 115.
	Intron	61, 65, 695, 449, 180, 59, 489.
ApCht7	Exon	291, 90, 232, 237, 179, 123, 309, 235, 190, 217, 295, 147, 185, 122, 201, 908.
	Intron	16014, 8517, 1564, 922, 85, 75, 365, 61, 67, 85, 62, 67, 59, 89, 569.
ApCht10	Exon	695, 282, 193, 182, 142, 135, 211, 133, 318, 135, 125, 185,142, 135, 211, 179, 363, 163, 204, 190, 452, 222, 112, 189, 227, 141, 129, 182, 53, 216, 305, 277, 188, 164, 91, 162, 125.
	Intron	57, 642, 91, 365, 91, 137, 95, 62, 883, 770, 65, 76, 63, 84, 64, 63,

		61, 85, 80, 65, 568, 90, 377, 68, 63, 57, 66, 64, 71, 57, 72, 59, 72, 67, 324, 3749.
ApENGase	Exon	1927, 224.
	Intron	442.

Supplementary Figure 1. Conserved regions in the glycoside hydrolase family 18 (GH18) domain of 5 *A. pisum.* Amino acid sequences of the catalytic domains of GH18 family enzymes were aligned using CLUSTALX software. Two and four catalytic domains of *ApCht7* and *ApCht10* were named as *ApCht7-1/2* and *ApCht10-1/2/3/4*, respectively. Boxed regions are the four conserved motifs represented by the sequences KxxxxxGGW (Mofit I), FDGxDLDWEYP (Mofit II), MxYDxxG (Mofit III) and GxxxWxxDxDD (Mofit IV).

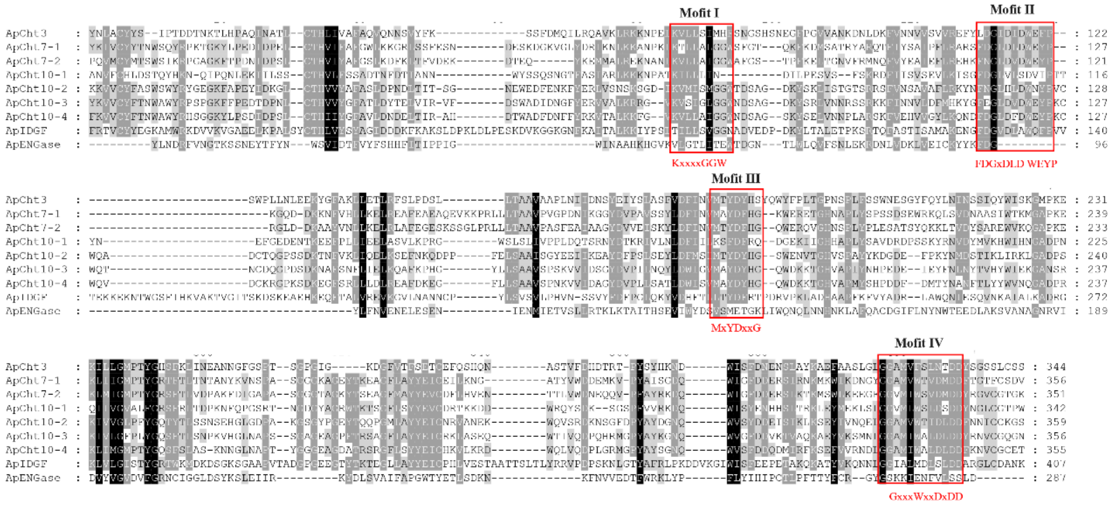

Supplementary Figure 2. Expression of the chitinase genes in response to temperature stress. Different lowercase letters are statistically different under same temperature stress in *A. pisum* ($p < 0.05$).

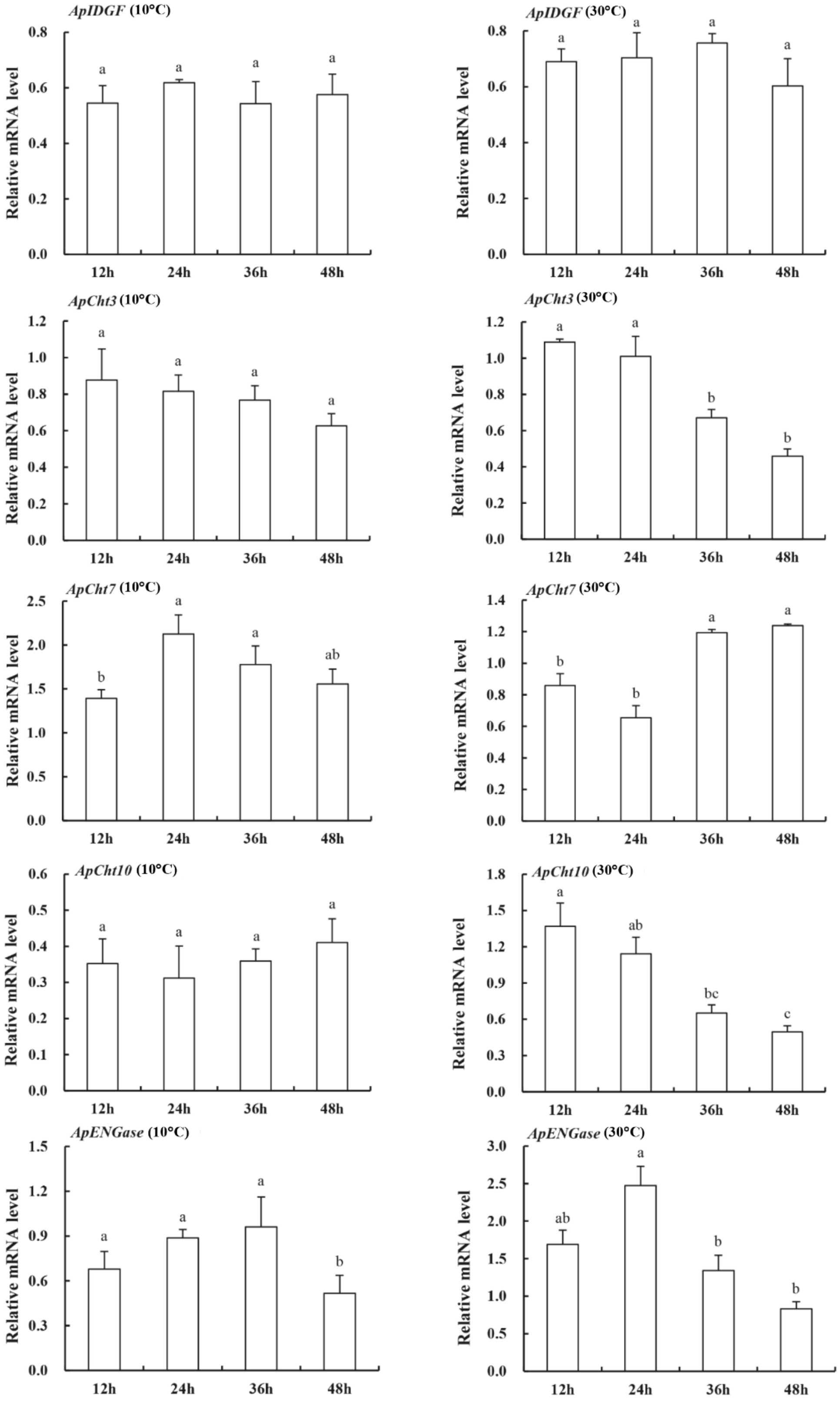

Supplementary Figure 3. Expression of the chitinase genes in response to insecticide stress. Different lowercase letters are statistically different under insecticide stress in *A. pisum* ($p < 0.05$).

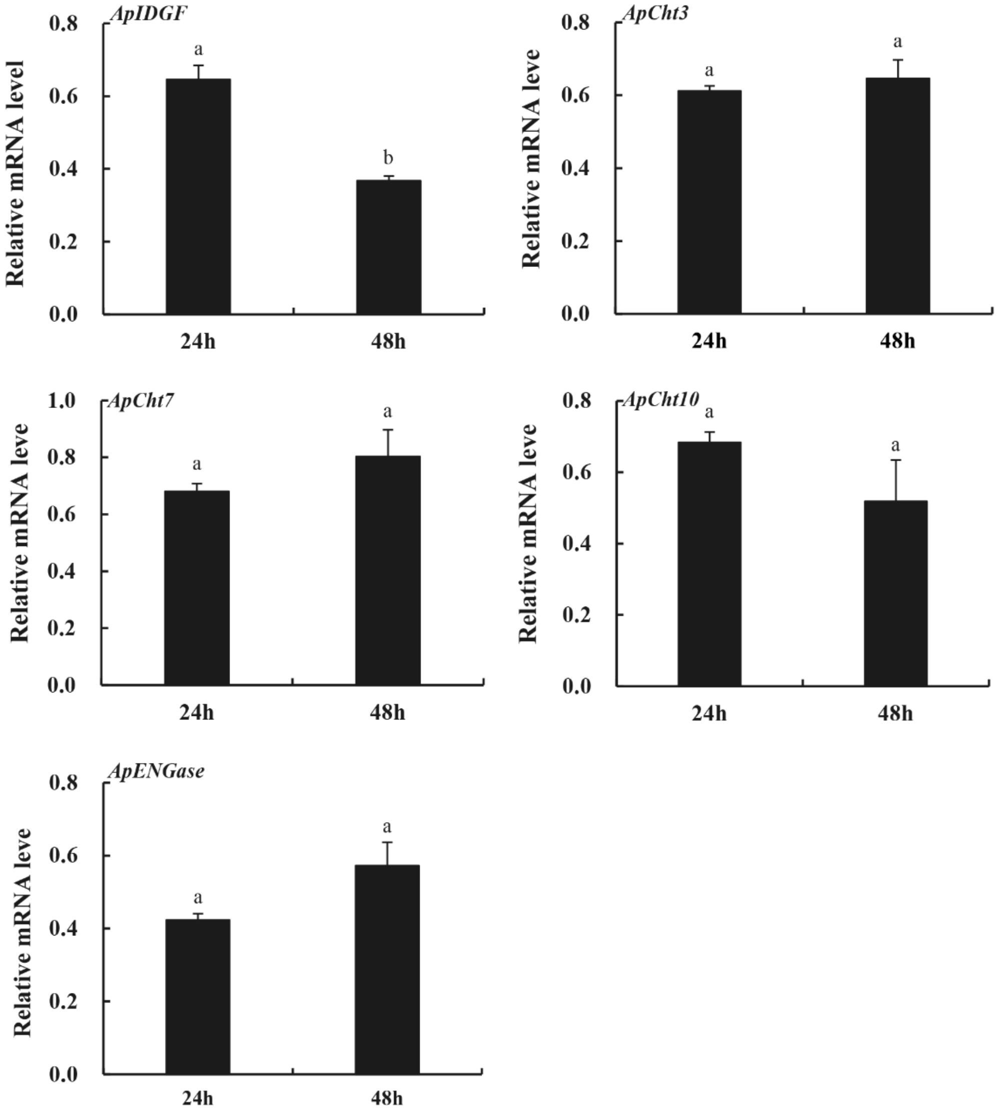

Supplementary Figure 4. Expression of the chitinase genes in response to 20E treatment. Different lowercase letters are statistically different under 20E stress in *A. pisum* ($p < 0.05$).

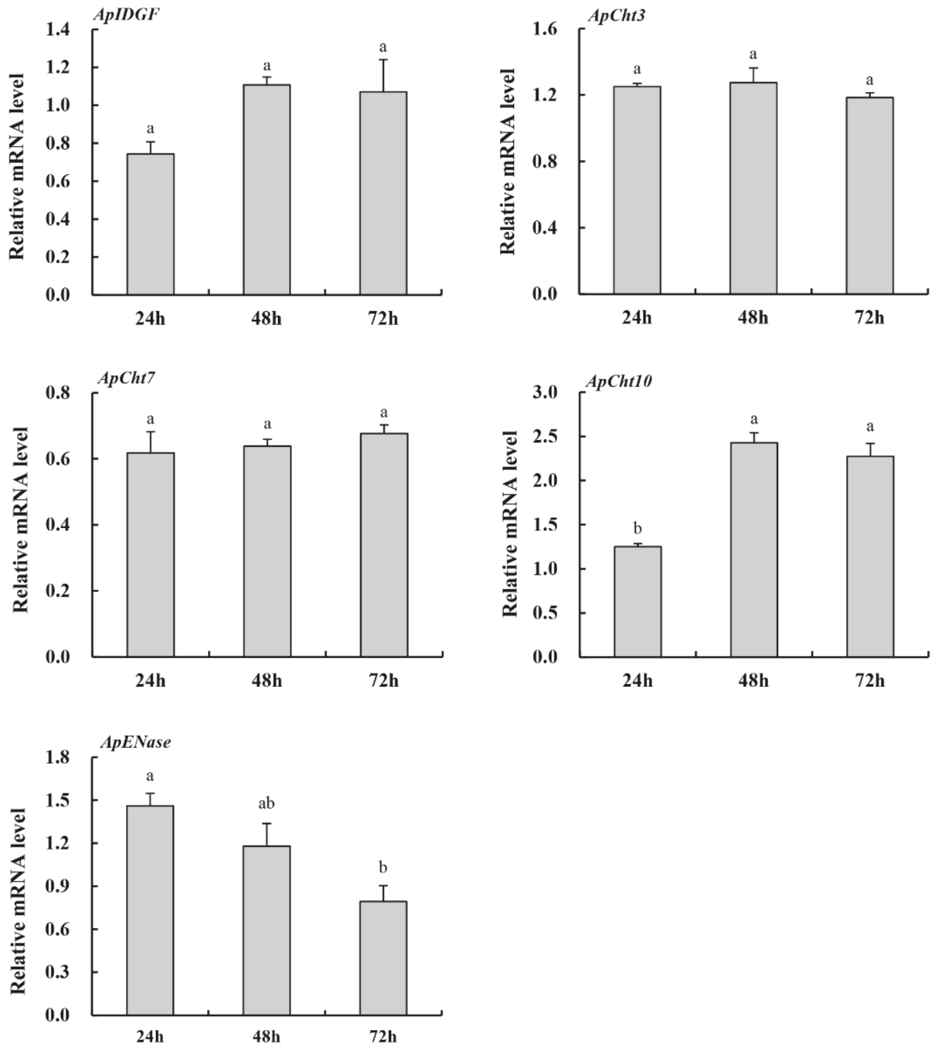

Supplement: Supplementary file 1 [file DataSheet1.pdf]
