# Supplementary material for: Effects of abiotic stresses on the expression of chitinase-like genes in Acyrthosiphon pisum
Source: Front Physiol. 2022 Nov 23;13:1024136. doi: 10.3389/fphys.2022.1024136 (PMC9727142; doi:10.3389/fphys.2022.1024136)

Supplementary Table 1. Information of insect chitinase proteins for phylogenetic tree construction

| Species | Protein | Accession number | Phylogenetic group |
| --- | --- | --- | --- |
| *Anopheles gambiae* | *AgCht2* | XP_315650 | Diptera |
|  | *AgCht4* | XP_315351 | Diptera |
|  | *AgCht5-1* | HQ_456129 | Diptera |
|  | *AgCht5-2* | HQ_456130 | Diptera |
|  | *AgCht5-3* | HQ_456131 | Diptera |
|  | *AgCht5-4* | HQ_456132 | Diptera |
|  | *AgCht5-5* | HQ_456133 | Diptera |
|  | *AgCht7* | XP_308858 | Diptera |
|  | *AgCht8* | XP_316448 | Diptera |
|  | *AgCht10* | XP_001238192 | Diptera |
|  | *AgCht11* | XP_310662 | Diptera |
|  | *AgIDGF2* | XP_001237925 | Diptera |
|  | *AgIDGF4* | XP_317398 | Diptera |
| *Drosophila melanogaster* | *DmCht2* | NP_477298 | Diptera |
|  | *DmCht4* | NP_524962 | Diptera |
|  | *DmCht5* | NP_650314 | Diptera |
|  | *DmCht7* | NP_647768 | Diptera |
|  | *DmCht8* | NP_611542 | Diptera |
|  | *DmCht9* | NP_611543 | Diptera |
|  | *DmCht10* | EAA46011 | Diptera |
|  | *DmCht11* | NP_572361 | Diptera |
|  | *DmIDGF1* | NP_477258 | Diptera |
|  | *DmIDGF2* | NP_477257 | Diptera |
|  | *DmIDGF3* | NP_723967 | Diptera |
|  | *DmIDGF4* | NP_727374 | Diptera |
|  | *DmIDGF5* | NP_611321 | Diptera |
|  | *DmIDGF6* | NP_477081 | Diptera |
| *Tribolium castaneum* | *TcCht4* | NP_001073567 | Coleoptera |
|  | *TcCht5* | NP_001034524 | Coleoptera |
|  | *TcCht6* | XP_967813 | Coleoptera |
|  | *TcCht7* | NP_001036035 | Coleoptera |
|  | *TcCht8* | NP_001038094 | Coleoptera |
|  | *TcCht9* | NP_001038096 | Coleoptera |
|  | *TcCht10* | NP_001036067 | Coleoptera |
|  | *TcIDGF2* | NP_001038092 | Coleoptera |
|  | *TcIDGF4* | NP_001038091 | Coleoptera |
|  | *TcENGase* | XP_969648.1 | Coleoptera |
| *Bombyx mori* | *BmCht1-1* | XP_004931749 | Lepidoptera |
|  | *BmCht2* | XP_004933352 | Lepidoptera |
|  | *BmCht5* | AAB47538 | Lepidoptera |
|  | *BmIDGF* | NP_001036847 | Lepidoptera |
| *Phenacoccus solenopsis* | *PsCht3-3* | MH686272 | Hemiptera |
|  | *PsCht5* | MH686266 | Hemiptera |
|  | *PsCht10* | MH686270 | Hemiptera |
|  | *PsIDGF* | MH686273 | Hemiptera |
| *Aphis gossypii* | *AgoCht3-2* | KAF0755855 | Hemiptera |
| *Apis mellifera* | *AmCht10* | XP_026299805 | Hymenoptera |
|  | *AmENGase* | XP_001121069 | Hymenoptera |
| *Bactrocera dorsalis* | *BdCht1* | MF926351 | Diptera |
|  | *BdCht2* | KF289944 | Diptera |
|  | *BdCht5* | KY681041 | Diptera |
|  | *BdCht7* | KY681042 | Diptera |
|  | *BdCht8* | KY426795 | Diptera |
|  | *BdCht10* | MK518061 | Diptera |
|  | *BdCht11* | KY426794 | Diptera |
|  | *BdIDGF1* | KY681043 | Diptera |
|  | *BdIDGF2* | KY681044 | Diptera |
|  | *BdIDGF3* | KY681045 | Diptera |
|  | *BdIDGF4* | KY681046 | Diptera |
|  | *BdIDGF6* | KY426796 | Diptera |
| *Nilaparvata lugens* | *NlCht5* | KM217113 | Hemiptera |
|  | *NlCht6* | KM217114 | Hemiptera |
|  | *NlCht7* | KM217115 | Hemiptera |
|  | *NlCht10* | KM217118 | Hemiptera |
|  | *NIIDGF* | KM217119 | Hemiptera |
| *Acyrthosiphon pisum* | *ApCht3* | XM_001952683 | Hemiptera |
|  | *ApCht7* | XM_001950345 | Hemiptera |
|  | *ApCht10* | XM_001943003 | Hemiptera |
|  | *ApIDGF* | [NM_001168671](https://www.ncbi.nlm.nih.gov/nuccore/NM_001168671) | Hemiptera |
|  | *ApENGase* | XM_001949910 | Hemiptera |

Supplementary Table 2. Primers for qPCR the genes of chitinase in *Acyrthosiphon pisum*

| Genes | Primers | Nucleotide sequence (5′-3′) |
| --- | --- | --- |
| *ApIDGF* | *ApIDGF*-F  *ApIDGF*-R | GGGTATCTCTACGTACGGCC  AAGGGTGAGAGATGTGGTGG |
| *ApCht3* | *ApCht3*-F  *ApCht3*-R | CGGCAAGGACGGTTTTGTAA  GAAAATACCATGGCGCCTCC |
| *ApCht7* | *ApCht7*-F  *ApCht7*-R | TACCTGAAGACATCGACCCG  AACTTTTGTGTGCCGAACGA |
| *ApCht10* | *ApCht10*-F  *ApCht10*-R | ACTGGTCCAAGATCCACTGG  GATACGTTTCGCAGCCACAT |
| *ApENGase* | *ApENGase*-F  *ApENGase*-R | ATGTTGACGGTGAAGCAGTT  TCCCTGAATGCCAAACTCCA |
| *EF1α* | *EF1α*-F  *EF1α*-R | CTGTGCTTATTGTCGCTGCT  TCGCTGTATGGTGGTTCAGT |
| *RPS20* | *RPS20*-F  *RPS20*-R | AAGTGTGTGCTCCGAGATGA  CAGCAATGACACCGGGTTC |

Supplementary Table 3. Lengths of each exon and intron of Chitinase gene in *Acyrthosiphon pisum*

| 基因  Gene | 类别  Structure | 从5’到3’长度  From 5’to 3’Length (5’-3’) |
| --- | --- | --- |
| *ApIDGF* | Exon | 553, 400, 171, 133, 269, 345. |
|  | Intron | 3381, 1134, 223, 644,1701. |
| *ApCht3* | Exon | 548, 215, 224, 136, 271, 166, 103, 115. |
|  | Intron | 61, 65, 695, 449, 180, 59, 489. |
| *ApCht7* | Exon | 291, 90, 232, 237, 179, 123, 309, 235, 190, 217, 295, 147, 185, 122, 201, 908. |
|  | Intron | 16014, 8517, 1564, 922, 85, 75, 365, 61, 67, 85, 62, 67, 59, 89, 569. |
| *ApCht10* | Exon | 695, 282, 193, 182, 142, 135, 211, 133, 318, 135, 125, 185,142, 135, 211, 179, 363, 163, 204, 190, 452, 222, 112, 189, 227, 141, 129, 182, 53, 216, 305, 277, 188, 164, 91, 162, 125. |
|  | Intron | 57, 642, 91, 365, 91, 137, 95, 62, 883, 770, 65, 76, 63, 84, 64, 63, 61, 85, 80, 65, 568, 90, 377, 68, 63, 57, 66, 64, 71, 57, 72, 59, 72, 67, 324, 3749. |
| *ApENGase* | Exon | 1927, 224. |
|  | Intron | 442. |

Supplementary Figure 1. Conserved regions in the glycoside hydrolase family 18 (GH18) domain of 5 *A. pisum.* Amino acid sequences of the catalytic domains of GH18 family enzymes were aligned using CLUSTALX software. Two and four catalytic domains of *ApCht7* and *ApCht10* were named as *ApCht7-1/2* and *ApCht10-1/2/3/4*, respectively. Boxed regions are the four conserved motifs represented by the sequences KxxxxxGGW (Mofit Ⅰ), FDGxDLDWEYP (Mofit Ⅱ), MxYDxxG (Mofit Ⅲ) and GxxxWxxDxDD (Mofit Ⅳ).


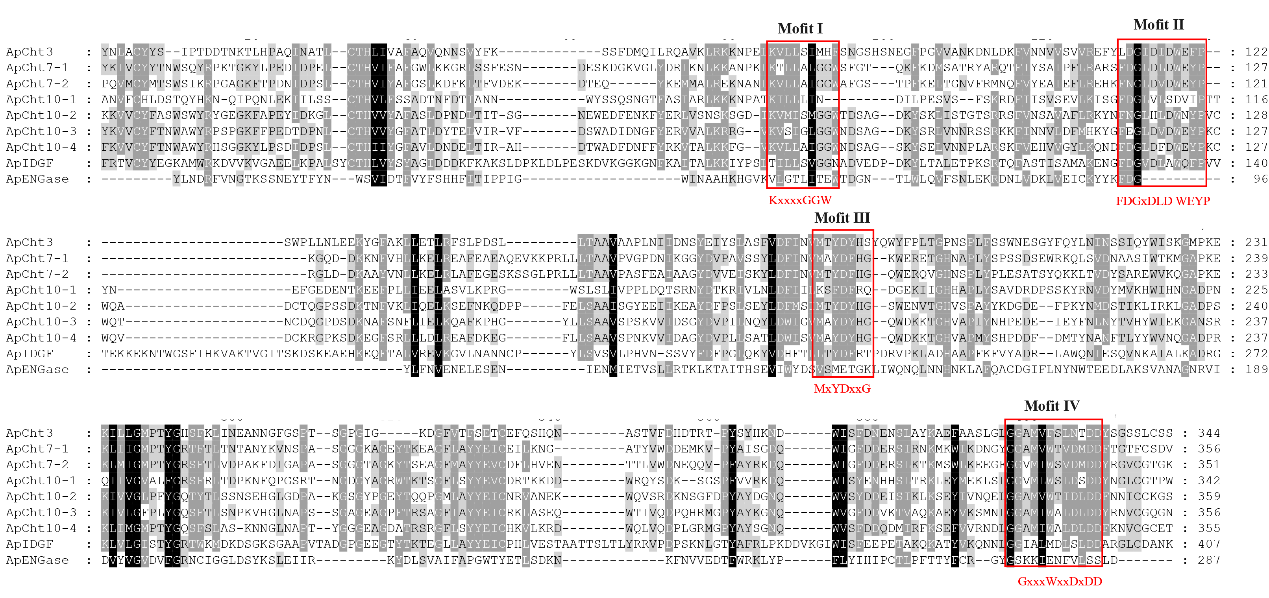

Supplement: Supplementary file 2 [file DataSheet1.docx]
